# Editorial: Physiology of Myelin Forming Cells, From Myelination to Neural Modulators

**DOI:** 10.3389/fncel.2019.00475

**Published:** 2019-10-22

**Authors:** Marion Baraban, Mauricio A. Retamal, Fernando C. Ortiz

**Affiliations:** ^1^Mechanics of Neuronal Development Laboratory, Institut de Biologie Paris-Seine—Developmental Biology Laboratory, CNRS UMR7622, Sorbonne Université, UPMC Univ Paris 06, Paris, France; ^2^Universidad del Desarrollo, Centro de Fisiologia Celular e Integrativa, Facultad de Medicina Clinica Alemana, Santiago, Chile; ^3^Department of Cell Physiology and Molecular Biophysics, Center for Membrane Protein Research, Texas Tech University Health Sciences Center, Lubbock, TX, United States; ^4^Mechanisms of Myelin Formation and Repair Laboratory, Instituto de Ciencias Biomédicas, Facultad de Ciencias de la Salud, Universidad Autónoma de Chile, Santiago, Chile

**Keywords:** oligodendrocyte, Schwann cell, oligodendrocyte precursor cell, myelin, myelin repair, axon physiology

Myelin is a specialized glial-derived membrane that enwraps axons enabling them for saltatory conduction of action potentials. It is organized in sheaths along the axonal compartment, separated by regions enriched in voltage-gated sodium channels—called *nodes of Ranvier*—where the action potential is regenerated. For many years myelin was considered a static structure, however, the idea of myelin coating as a rigid insulator of axons has been challenged in the last decades. Indeed, both central and peripheral myelin forming cells, namely oligodendrocytes and Schwann cells, interact actively with the surrounding neuronal and non-neuronal cells supporting many processes such as neuronal activity and metabolic supply. In addition, evidences provided by several research groups indicate that myelin synthesis is not only restricted to development, but it is also contributing to the remodeling in adulthood and the repair under pathological conditions. The aim of the present Research Topic is to group articles, reviews and technical reports exploring different aspects of the myelinating cells function in health and disease.

Intercellular communication between myelinating glial cells and the rest of the cellular components of the nervous tissue is essential to maintain CNS homeostasis. This function is mainly based on the ability of glial cells to be metabolic and electrically coupled between themselves and neurons, participating in trophic support and synchronization of the glial network. This communication is established by devoted channels constituted by connexins or pannexins which are permeables to ions and small molecules. In this regard, Cisterna et al. summarize the organization and the functional role of gap junction channels constituted by connexins in Schwann cells. They further report the consequences of the mutation of genes encoding connexin 32, leading to Charcot Marie Tooth X-linked form 1 disease (Cisterna et al.). Vejar et al. review the functional evidence of electrical and metabolic coupling through connexin- and pannexin-based channels within and between oligodendrocytes in the central nervous system. Afterward, the authors focus on the interaction and function of oligodendrocytes/astrocytes coupling, a well-known process that provides metabolic support to axons.

Subsequently, two technical reports illustrate the use of advanced techniques based on optogenetics and optical sensors, applied to the study of oligodendroglia physiology (Looser et al.; Ortolani et al.). Looser et al. optimize the combination of electrophysiological recordings and imaging of an ATP biosensor in the fully myelinated optic nerve. The ATP sensor was delivered by reliable intravitreal injections of adeno-associated virus vectors which make this protocol adaptable to any genetically encoded sensor in any mutated mice. This method will permit to gain knowledge on the metabolism and homeostasis of this myelinated nerve in both physiological and pathological conditions. Moreover, Ortolani et al. established a protocol to perform photo-stimulation of GABAergic neurons *in vivo*, in the awake mouse pups. The authors confirmed that GABAergic synaptic inputs have no effect on OPC proliferation of the somatosensory cortex during development. Remarkably, this framework was optimized at an early post-natal stage, during the critical period of intense myelination. This might be readily adapted to investigate activity-dependent myelination occurring in other brain areas of the mouse.

The next section recapitulates some of the key aspects of myelination in the central nervous system. While it is known that oligodendrocytes myelinate specific axons, the complex rules governing the correct targeting of CNS myelination only begin to be understood. Almeida highlights the molecular and cellular codes that possibly target the myelination of the appropriate axon. After the initial step of targeted myelination, one can ask how the CNS myelination pattern changes during adulthood. Williamson and Lyons provide an overview of the recent findings on myelination dynamics and remodeling, specially they discuss the interesting results obtained by recent longitudinal imaging studies, concerning the apparent stability of the myelination patterns along the axon over lifetime.

Many human neurologic conditions have been associated with glial cells dysfunction or destruction in the CNS. Among them multiple sclerosis (MS) represents the archetypical example of a demyelinating disease, being the second cause of disabilities for the young adult population and more than 2 million people having the condition worldwide. By using the experimental autoimmune encephalomyelitis (EAE) MS model, Osorio-Barrios et al. examine the contribution of dopamine receptor D5 pathway on T-Cells, knowing to play a key role on the early stages of the demyelination insult in MS. It is known that after this initial demyelinated stage there is a spontaneous remyelination process that normally remains incomplete. This has led to the develop of many studies trying to shed light on the remyelination mechanisms, aiming to improve this spontaneous repair process. One strategy to increase the efficiency of remyelination might be to produce additional myelinating cells with the help of neighboring cells. In this regard, Silva et al. demonstrate that pericytes, central components of the vascular niche, influence neural stem cells toward the generation of oligodendrocytes. In this same line, to identify molecular factors involved in adult oligodendrogenesis is central in the search of myelin regeneration strategies. Laouarem and Traiffort highlight the importance of the neural morphogen hedgehog and its pathway during myelin repair, as well as providing an overview on the role of this signaling molecule during oligodendrogenesis at early developmental stages. Recent studies have pointed out oligodendroglia abnormalities as a contributing factor on complex pathologies such as the autistic spectrum disorder (ASD). To investigate in further details those abnormalities, Graciarena et al. characterize myelination and oligodendroglia population of different brain areas in a murine ASD model induced by prenatal exposition to valproic acid. In addition, Shen et al. summarize the contribution of adenosine signaling to both oligodendrocyte homeostasis and myelination in the context of the ASD.

Finally, a collection of reviews and articles concentrates on summarizing the miscellaneous functions of myelinating glial cells. Couve and Schmachtenberg describe the dental pulp system in human, focused on its innervation, the myelinating and the non-myelinating Schwann cells. Interestingly, they review the striking plasticity of those two types of Schwann cells preserving the dental pulp innervation, after an acute nerve injury caused by caries as well as the long-term changes related to aging. Fontenas and Kucenas introduce and depict an exceptional novel population of glial cells called motor exit point (MEP) glia. MEP glial cells are located at the transition zone in the interface between the peripheral and the central nervous systems, being able to myelinate motor neurons at that particular axonal region. The authors report the main characteristics of this recently described glia population based on their *in vivo* studies in zebrafish, emphasizing the importance of *in vivo* experimental models to study cellular dynamics (Fontenas and Kucenas). The next article examines one particular aspect of the microglia/oligodendroglia interaction: Thomas and Pasquini concentrate on the role of microglia derived-galectin-3 in oligodendrocyte differentiation and how this signaling molecule can foster remyelination. Finally, Nayak et al. bring our attention to the intracellular domain (ICD) of the NG2 proteoglycan, a molecular hallmark of oligodendrocyte progenitors, by showing that this domain induce mRNA translation mainly through the mTOR pathway. They suggest that the intracellular domain of NG2 could accelerate cell cycle kinetic in OPC and hypothesize that NG2 ICD might play a role also in tumor progression.

Summarizing, this Research Topic present original articles and updating reviews on the cellular and molecular mechanisms of the classical function associated with myelin forming cells, namely myelin formation and repair processes. But more interestingly, the topic also recapitulates evidence on, possibly unexpected, new functions (i.e., preserving dental pulp cytoarchitecture); new cellular interactions, such as the role of pericytes on remyelination; and even a new population of myelinating glia (i.e., MEP glia). By organizing this topic, we expect to provide a unique and broad overview on myelinating cells function in health and disease, encouraging to the community to develop more investigation on this very active field.

## Author Contributions

MB and FO conceived the major ideas developed in the manuscript. MB wrote the original manuscript which was edited and corrected by all the authors. MB, MR, and FO approved the final manuscript.

### Conflict of Interest

The authors declare that the research was conducted in the absence of any commercial or financial relationships that could be construed as a potential conflict of interest.

